# Application of the Theory of Planned Behaviour to inform development of a Dissemination and Implementation science training for nutrition practitioners

**DOI:** 10.1017/S1368980023002525

**Published:** 2023-12

**Authors:** Ayron E Walker, Daniel Totzkay, Samantha E Scarneo-Miller, Elizabeth A Claydon, Melissa D Olfert

**Affiliations:** 1 Nutrition and Health Care Management, Beaver College of Health Sciences, Appalachian State University, 1179 State Farm Rd, Boone, NC 28607, USA; 2 Division of Animal and Nutritional Sciences, Davis College of Agriculture, Natural Resources and Design, West Virginia University, Morgantown, WV, USA; 3 Department of Communication Studies, Eberly College of Arts and Sciences, West Virginia University, Morgantown, WV, USA; 4 Division of Athletic Training, School of Medicine, West Virginia University, Morgantown, WV, USA; 5 Department of Social & Behavioral Sciences, School of Public Health, West Virginia University, Morgantown, WV, USA

**Keywords:** Dissemination and Implementation science, Theory of Planned Behaviour, Nutrition, Nutrition interventions, Training development

## Abstract

**Objective:**

To determine nutrition practitioners’ attitudes, behavioural control and normative beliefs to best inform the development and formulation of a nutrition-specific Dissemination and Implementation (D&I) science training.

**Design:**

A cross-sectional survey aimed to assess Theory of Planned Behaviour (TPB) constructs and intention to use D&I science. A validated TPB questionnaire assessed constructs including perceived behavioural control, subjective, injunctive and descriptive normative beliefs, attitudes and intention to use D&I science. For analysis, Spearman’s *ρ*, Kruskal–Wallis and Steel–Dwass tests were conducted for quantitative variables.

**Setting:**

Online, 26-item Qualtrics survey.

**Participants:**

Cross-sectional sample of members (*n* 70) affiliated with the Society for Nutrition Education and Behaviour listserv.

**Results:**

The major finding from this study was a significant positive correlation between perceived behavioural control score and intention (*r* = 0·315, *P* = 0·0119).

**Conclusions:**

D&I training interventions could formulate learning and teaching strategies to target perceived behavioural control (self-efficacy, knowledge and ability) to enhance intention. For example, application and experience-based learning techniques trainings could be strategies to increase knowledge and abilities.

At its core, education and training initiatives provide continued education for nutrition professionals to alter behavioural intention^(1)^. Behavioural intention describes the beliefs, assumptions and personal factors that influence a given behaviour^(2)^. Certain theories postulate that factors such as attitudes, perceived behavioural control, and normative beliefs influence the likelihood of behavioral intention change^(2)^. For example, the three theoretical constructs identified previously are the motivational factors from the Theory of Planned Behaviour (TPB) that determined the likelihood of specific behaviour change^(2,3)^. TPB assumes a causal linkage between normative and behavioural beliefs to intentions directed by attitudes, perceived behavioural control and subjective norms and has framed numerous health behaviour change studies and educational messaging^(2,3)^. TPB is applied to many and varying health behaviours studies that are previously reviewed and resulted positively for rigour and effectiveness^(4,5)^. The prevalence of TPB usage in public health and its applicability to nutrition professional education is in part due to its ease of operationalisation in developing, analysing and measuring behaviours and interventions^(4,5)^.

The capability to effectively predict and describe factors that influence behavioural intention is important to research in health including nutrition, physical activity and sex education and assists to develop interventions to change specific behaviours^(4)^. For example, Asare studied condom use among college students using the TPB and reported behavioural intention significantly predicted condom use^(6)^. Additionally, other interventions including communicating health messages to clients^(7,8)^, and health behaviour education interventions^(6,9,10)^, that utilised the TPB see changes in attitudes, perceived behavioural control, normative beliefs and intention among participants. Hence, it is necessary to understand the current knowledge, self-efficacy and attitudes of a study population such as nutrition professionals when designing an educational training to ensure that messaging is directing behavioural intention.

Unfortunately, health education and interventions today are not always theory-based, which can lead to misconceptions and limited dissemination of evidence-based information^(1)^. Misconceptions, about behaviours due to inconsistent or lack of information, can lead to an absence of individual intention to participate in activities^(7)^. Frequently, individuals do not have a clearly defined behavioural response to a particular information^(11)^. When a population has limited experiences or education with the intended behavioural change, then their attitudes, beliefs and knowledge must be shaped into a new behaviour^(11)^. This response-shaping process is important when new information is constantly evolving or there is a limited understanding or experience with the information within the message^(11)^, which is especially relevant for emerging science and updated professional practice. A potential response-shaping process is targeted, theory-based trainings for populations with low self-efficacy, attitudes and normative beliefs. For example, nutrition practitioners have a limited understanding and knowledge of Dissemination and Implementation (D&I science), which demands education and messaging to be formulated in a way that will change behaviour^(12)^.

D&I science is the study of the integration and translation of research findings into health practices or interventions^(13,14)^. D&I science describes two components: dissemination, which is defined as the active approach of spreading evidence-based interventions to the target audience, and implementation, which is defined as the process of putting to use or integrating evidence-based interventions within a setting^(15)^. The historical roots of D&I science stem from the diffusion of innovations theory^(16)^ and the agricultural extension model^(17)^, which provided the building blocks for evidence-based public health research that led to D&I science foci in cancer, mental health and substance use^(13,17)^. In recent years, specific fields in public health have increasingly recognised the need to use D&I science to enhance rigour of interventions (policies, programmes, education, information and knowledge translation). Furthermore, evaluations of current D&I trainings to increase capacity and usage of D&I science in health highlight improvements in knowledge, confidence and changes to research practice (incorporation of D&I science into research)^(18–26)^. A recent review describes how more involvement in D&I-focused research has led to the evaluation of adaptations to programme implementation, which has increased the systematic documentation and reporting to improve replication and patient-centred outcomes^(27)^. Yet, a recent systematic review highlighted the current contexts of D&I science trainings, in which zero were explicitly focused on nutrition^(20)^. Without proper training, nutrition practitioners and researchers have an incomplete understanding about what D&I science is, which hinders their usage and involvement and impacts the translation of health information and interventions to populations^(12,28–31)^. Therefore, reviews suggest that nutrition-specific trainings are needed to increase the use of D&I science among nutrition practitioners and researchers to improve nutrition interventions, dietary patterns and nutrition-related outcomes^(30)^. Without such training, nutrition interventions will continue to see implementation challenges such as the absence of sustained, effective and appropriately adapted nutrition educations, programmes or policies^(12,30)^. Thus, the purpose of this research study was to determine how nutrition practitioners’ attitudes, behavioural control and normative beliefs influenced their intention to use D&I science in their professional practice to best develop and formulate a TPB-based nutrition-specific D&I training.

## Materials and methods

### IRB approval

This study was approved by BLINDED FOR REVIEW Institutional Review Board (protocol # BLINDED FOR REVIEW).

### Theoretical framework

**Fig. 1:**
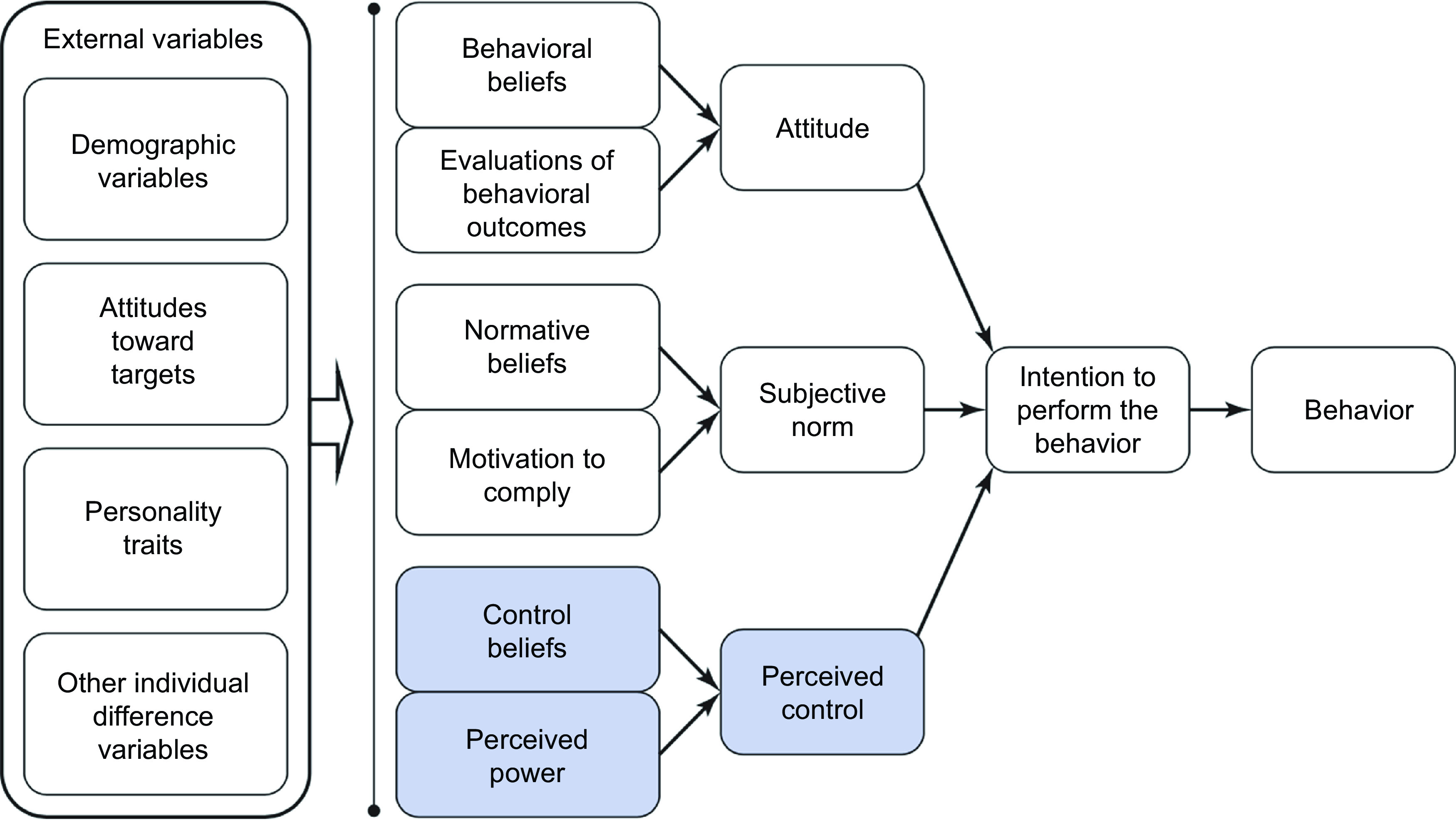
Theory of Planned Behaviour. ^a^Shaded areas represent the theory of reasoned action. ^b^Figure from: Montaño & Kasprzyk (2014)^(3)^

TPB (Fig. 1) was developed in the 1980s by Icek Ajzen using particular constructs to predict human behaviour^(2)^. This theory postulates that attitude towards a given behaviour, normative beliefs about said behaviour and perceived control over that behaviour are all predictors of behavioural intention^(2)^.

Behavioural intention, the first construct of the TPB, is defined as the motivational factor to achieve a specific behaviour^(2)^. The stronger the intention, the more likely it is that an individual will complete the intended behaviour^(6)^. The attitude towards the behaviour, the second construct of the TPB, describes someone’s positive or negative thoughts or reactions towards the given behaviour^(2)^. The third construct of the TPB, normative beliefs, defines the social pressures or influences someone experiences about the given behaviour^(2)^. The final construct, perceived behaviour control (i.e. knowledge, learning autonomy) refers to someone’s ability to and knowledge of a given behaviour and how that influences their capacity to perform^(2)^.

### Participants and recruitment

This study was a cross-sectional study to identify current attitudes, perceived behavioural control and normative beliefs among nutrition practitioners (including clinical and private practice dietitians, researchers, outpatient nutrition educators, nutrition and dietetic students, academics (professors, researchers)) to understand what factors would most likely assist in increasing intention to use D&I science. To recruit participants, a weekly email was sent to the Society for Nutrition Education and Behaviour membership listserv (referred to as SNEEZE), which includes roughly 1000 members from a variety of different occupancies such as clinical dietetics, higher education, private sector and nutrition educators at a variety of locations such as State Cooperative Extension, public health department, private practice clinics and outpatient settings. An initial email was sent to the SNEEZE listserv in early September 2020 containing a Qualtrics survey link (Qualtrics, Provo, UT, 2021). A reminder email was sent every 2 weeks until the beginning of November 2020. The survey remained active from September 2020 to November 2020.

### Hypothesis testing

For non-parametric testing, four hypotheses were tested and listed below.

1. Attitudes toward behaviour (use of D&I science), subjective norms, descriptive norms, injunctive norms (described as normative beliefs) and perceived behavioural control will be positively correlated with intention.
2. Higher expert score (proficient and expert level) will be positively correlated with attitudes, normative beliefs and perceived behavioural control.
3. Training recipients (having received training) will be positively correlated with higher perceived behavioural control.
4. Perceived behavioural control will be positively correlated with attitudes and normative beliefs.

### Evaluation survey

The questionnaire was constructed based on an existing TPB survey development manual^(32)^ and validated measurements^(33)^. The survey development manual was designed to assist researchers in health services to produce consistent and effective questionnaires to measure TPB constructs^(32)^. These TPB constructs have been validated and used in a variety of different settings and populations to predict behavioural intention and ultimately create interventions to change behaviours^(4)^.

Before accessing the online survey, participants were required to read and accept informed consent via two questions. Then, participants would complete twenty-four questions and one question to gather contact information for gift card recipients. One survey question assessed for previous training (‘have you had any prior training in Dissemination and Implementation science?’) and another survey item measured self-ranked D&I expertise (categories described below). The remaining twenty-two questions assessed the constructs of the TPB through a 7-point Likert scale (1 – strongly disagree and 7 – strongly agree) and open-ended questions. The behaviour in question was the use of D&I science. The survey was reviewed and approved by all authors prior to survey dissemination. Evaluation survey is provided as online supplementary material.

### Measurement scoring

#### Expert ranking

Participants categorised their perception of D&I experience. Categories and definitions included inexperienced (never heard of D&I science before); novice (a person new to or inexperienced in the D&I field, i.e. limited understanding of D&I science); beginner (a person just starting to learn D&I science, i.e. have some knowledge base, e.g., could vaguely define D&I science); proficient (competent or skilled in doing or using something, i.e. have conducted D&I research and/or attended webinars/trainings/conference sessions and could explain to others the many attributes of D&I science) or expert (displaying special skill or knowledge derived from training or experience (i.e. have numerous experiences conducting D&I research and/or attended many webinars/trainings/conference sessions and could confidently explain/teach others the many attributes of D&I science). Responses were coded as ordinal variables (with five distinct levels) for quantitative analysis.

#### Participant training

For training scores, participants were asked to describe if they had ever received D&I training in the past. Qualitative responses were manipulated to binary categorical variables (yes or no) for analysis. Additionally, descriptions of received D&I trainings were grouped to provide further information.

#### Theory of Planned Behaviour constructs score

Each construct (i.e. attitudes, normative beliefs (subjective, descriptive, injunctive), perceived behavioural control and intention to use) was developed and measured based on previous validated TPB survey tools and measurements^(32,33)^. Each survey question directly measured each construct. Survey responses to each construct were averaged for an overall mean score and were used as continuous variables for quantitative, non-parametric analysis.

#### Analysis

Quantitative data were analysed in JMP software (version pro 14, SAS Institute Inc.) and included descriptive statistics, frequency analysis and non-parametric measures. Non-parametric measures are statistical methods in which the data are not assumed to come from prescribed models that are determined by a small number of parameters or do not meet all statistical assumptions needed for parametric testing. This data did not meet all statistical assumptions for parametric testing (e.g. multivariable regression). Therefore, Spearman’s *ρ* tests were used to determine the strength and direction of correlations between TPB measures to understand relationships between TPB constructs. Kruskal–Wallis tests can determine how categorical independent variables relate to a continuous variable; however, this test cannot tell you which specific groups are statistically significant (an omnibus test statistic). For this study, Kruskal–Wallis tests were used to understand whether TPB measures (continuous variables) differed based on expert ranking and training. Since Kruskal–Wallis tests are an omnibus test statistic, Steel–Dwass tests were used to pairwise compare categories in expert rankings and participant training with TPB measures.

## Results

### Demographics

Table 1 describes descriptive statistics from quantitative analysis. The demographics of the participants (*n* 70) are similar to the clinical dietetics field^(34)^, yet they are not entirely representative or generalisable of all nutrition practitioners. Additionally, categorised open-ended questions on individual’s job or place of work resulted in most respondents worked in academics (*n* 31) as a professor, researcher or student. Many participants worked within outpatient nutrition education interventions (*n* 18), and other survey answers included clinical dietitians (*n* 9) (in-patient, outpatient and private practice) and administration (*n* 4). Furthermore, respondents could elect to describe the variety of D&I science trainings they received. In which, many (*n* 15) described receiving training during graduate school seminars, national conferences (*n* 7), grant writing workshops (*n* 12) and work experience (*n* 4) such as hospital administration or Cooperative Extension. Only two participants reported receiving explicit D&I science training including the Training Institute for D&I Research in Cancer or Training Institute for D&I Research in Health^(35)^.

**Table 1:** Participant demographics

Variables	Subcategories	*n*	%	Mean	sd	Min–Max
Gender	Males	2	2·9			
	Females	68	97·1			
Age (years)				42	13	25–75
Hispanic	Hispanic	3	4·3			
	Non-Hispanic	67	95·7			
Race	White/Caucasian	60	87·1			
	Hispanic	1	1·4			
	Black/African American	2	2·9			
	Asian/Asian American (East Asian)	1	1·4			
	Asian/Asian American (South Asian)	1	1·4			
	Middle Eastern	2	2·9			
	Other/preferred terminology	2	2·9			
Education	College – no degree	1	1·4			
	Bachelor’s degree	3	4·3			
	Master’s degree	39	55·7			
	Doctorate (e.g. PhD, EdD)	27	38·6			
Registered dietitian nutritionist	Yes	53	75·7			
	No	17	24·3			
Participant training	Yes (received training)	26	37·1			
	No (have not received training)	44	62·9			
Expert ranking	Inexperienced	13	18·6			
	Novice	16	22·8			
	Beginner	21	30·0			
	Proficient	18	25·7			
	Expert	2	2·9			

### Non-parametric measures

Table 2 reports quantitative analysis from the Spearman’s *ρ* analysis to test observed correlations among TPB variables. Significant positive correlations were shown between several independent and dependent variables including perceived behavioural control and intention score (*P* = 0·0119) and attitude (*P* = 0·0074); perceived behavioural control and subjective normative belief (*P* = <0·0001), injunctive normative belief (*P* = 0·0017) and descriptive normative belief (*P* = 0·0006).

**Table 2:** Correlations between TPB measures among participants

Variable	By variable	Spearman’s *ρ* (*n* 61)	Prob > |ρ|
Attitude score	Intention score	0·1387	0·2520
Subjective belief score	Intention score	0·1613	0·1921
Subjective normative belief score	Attitude score	0·2717	0·0261*
Injunctive normative belief score	Intention score	0·1586	0·2035
Injunctive normative belief score	Attitude score	0·2845	0·0206*
Injunctive normative belief score	Subjective normative belief score	0·7753	<0·0001*
Descriptive normative belief score	Intention score	0·1762	0·1537
Descriptive normative belief score	Attitude score	0·3074	0·0114*
Descriptive normative belief score	Subjective normative belief score	0·6619	<0·0001*
Descriptive normative belief score	Injunctive normative belief score	0·6955	<0·0001*
Perceived behavioural control score	Intention score	0·3150	0·0119*
Perceived behavioural control score	Attitude score	0·3343	0·0074*
Perceived behavioural control score	Subjective normative belief score	0·5286	<0·0001*
Perceived behavioural control score	Injunctive normative belief score	0·3945	0·0017*
Perceived behavioural control score	Descriptive normative belief score	0·4238	0·0006*

TPB measure, Theory of Planned Behaviour measures; Prob > |ρ|, probability of obtaining a Spearman’s *ρ* value greater than the one shown.

* Signifies statistical significance.

Table 3 reports quantitative analysis from the Kruskal–Wallis test analysis to test observed correlations among variables. Significant positive correlations were shown between attitudes and training (*P* = 0·0022); perceived behavioural control and expert score (*P* = 0·0126) and attitudes and expert score (*P* = 0·0025).

**Table 3:** Correlations between TPB measures, participant training and expert ranking

Variable	By variable	Chi-square	DF (*n* 61)	Prob > ChiSq
Subjective normative belief score (TPB measure)	Participant training	0·8562	1	0·3548
Injunctive normative belief score (TPB measure)	Participant training	0·5026	1	0·4784
Descriptive normative belief score (TPB measure)	Participant training	0·0580	1	0·8097
Perceived behavioural control score (TPB measure)	Participant training	1·1196	1	0·2900
Attitudes score (TPB measure)	Participant training	9·3410	1	0·0022*
Intention score (TPB measure)	Participant training	1·5740	1	0·2096
Subjective normative belief score (TPB measure)	Expert ranking	5·3793	4	0·2505
Injunctive normative belief score (TPB measure)	Expert ranking	5·5505	4	0·2353
Descriptive normative belief score (TPB measure)	Expert ranking	5·2821	4	0·2596
Perceived behavioural control score (TPB measure)	Expert ranking	12·7495	4	0·0126*
Attitudes score (TPB measure)	Expert ranking	16·4635	4	0·0025*
Intention score (TPB measure)	Expert ranking	4·9135	4	0·2963

TPB measure, Theory of Planned Behaviour measures; Prob > ChiSq, probability of obtaining a chi-square value greater than the one shown.

* Signifies statistical significance.

Table 4 reports the pairwise comparisons from the Steel Dwass. A significant positive correlation was identified between a higher training score (receiving a training) and a higher attitude score (*P* = 0·0023). Additionally, the pairwise comparison suggests higher levels of expertise correspond to attitudes and specifically in the proficient group when compared with the inexperienced group (*P* = 0·0016). Lastly, higher levels of expertise correspond to higher perceived behavioural control score (*P* = 0·0445), specifically compared between proficient and beginner groups.

**Table 4:** Steel–Dwass test comparison between expert ranking, TPB measures and previous participant training

Perceived behavioural control (TPB measure) – expert ranking					
Level	Level	Mean difference	*P*	Lower CL	Upper CL
Proficient	Beginner	9·69 659	0·0445*	0·0000	0·4055
Expert	Beginner	8·84 211	0·3055	.	.
Expert	Novice	7·08333	0·3303	.	.
Proficient	Novice	6·27 451	0·3103	−0·0541	0·3075
Proficient	IE	5·87 647	0·3297	−0·0572	0·3254
Expert	Proficient	5·86 765	0·6172	.	.
Expert	IE	5·10 000	0·3565	.	.
Beginner	IE	0·41 667	0·9999	−0·3254	0·2683
Beginner	IE	−3·28 158	0·8603	−0·3483	0·1625
Beginner	Novice	−3·63 860	0·8264	−0·3417	0·1759
Attitudes (TPB measure) – expert ranking					
Proficient	IE	12·3868	*P*	0·0526	0·4595
Proficient	Beginner	11·2976	0·0016*	0·0000	0·2703
Proficient	Novice	9·1493	0·0166*	0·0000	0·2595
Beginner	IE	3·9853	0·0561	−0·1144	0·24 512
Novice	IE	3·1370	0·7849	−0·1372	0·30 368
Expert	IE	1·4423	0·8590	.	.
Expert	Beginner	1·0952	0·9929	.	.
Expert	Novice	0·2813	0·9995	.	.
Beginner	Novice	0·0000	1·0000	−0·1671	0·1671
Previous participant training – attitudes **(**TPB measure)					
No training	Training	−15·2972	0·0023*	−0·1823	−0·0299

CL, confidence level; IE, inexperienced; TPB, Theory of Planned Behaviour.

* Signifies significance.

## Discussion

TPB is a broadly used and valuable theory for intervention development and behaviour change^(32)^. It is essential to apply theory when developing a training intervention to ensure appropriate, targeted behaviour change. The purpose of this study was to determine which TPB constructs were correlated with the intention to use D&I science among nutrition practitioners to provide theory-based foundations to inform the development of a training. D&I science and strategies are new approaches to the field of nutrition research and intervention science^(28,30,36)^, which makes it critical to understand intentions, attitudes, self-efficacy and normative beliefs prior to development of a training intervention to ensure strategies are appropriate and likely to affect behaviour. Additionally, without proper training, implementation challenges (sustainability, adaptability, reach, replicability) in nutrition interventions could persist influencing communities’ access to health information^(30)^. Therefore, the results from this study provide potential foundations for future D&I science trainings in nutrition.

For instance, the findings from this study highlight that perceived behavioural control was positively correlated with intention to use D&I science. These findings are congruent with previous studies that suggest perceived behavioural control is a strong predictor of intention to use among health professionals^(28,37)^. Interestingly, research suggests that perceived behavioural control can act as a moderator to intention and may be the essential construct to address in behaviour interventions^(5)^. While not all TPB constructs were correlated with intention (not supporting our hypothesis), the correlation between perceived behavioural control and intention suggests that training strategies could focus on providing nutrition-specific D&I information to target knowledge and potentially intention.

Likewise, research demonstrates that a population’s attitudes and normative beliefs influence the effectiveness of health interventions^(6)^. According to Asare and colleagues, perceived behavioural control can positively change in tandem with attitudes by utilising educational strategies that target populational beliefs, motivation and knowledge^(6)^. For example, shifting nutrition practitioners’ D&I science attitudes could positively affect perceived behavioural control. To do so, future training could conduct elicitation activities^(2,9)^, prior to the training implementation. Elicitation activities assist in identifying current perceptions (attitudes, knowledge and beliefs) about information to tailor the learning environment to different ideas to encourage dialogue and behavioural change. For example, the nutrition-specific D&I training could utilise elicitation activities to develop small groups that hold similar attitudes, knowledge or beliefs for guided discussions about D&I science.

In addition, attitudes and perceived behavioural control were correlated with a higher likelihood of being an expert in D&I (in congruent with our hypothesis). These findings align with Croce and colleagues’ findings, which describe how perceived behavioural control dictated expertise among participants^(39)^. Additionally, articles recently analysed in a scoping review concluded that the extent of D&I knowledge predicted whether stakeholders would recognise themselves or colleagues as experts^(30,40)^. This suggests that increasing knowledge could increase nutrition D&I science experts.

Yet, to create more experts in the field and increase knowledge, Walker and colleagues discussed the need to develop and provide a nutrition-specific D&I training for nutrition practitioners. Yet, there are limited nutrition-specific D&I trainings^(30)^ and many barriers to utilisation of broader D&I science trainings including enrolment timelines, strict eligibility criteria and low acceptance rates^(20)^. This highly selective nature of the current D&I science trainings could explain why over 60 % of our survey participants stated never receiving any D&I training, and only 28·6 % reported being proficient or an expert. Therefore, creative approaches to implementation could increase the number of available nutrition-specific D&I science trainings. For example, implementation approaches could utilise online platforms to implement learning material, which could encourage flexibility for participant involvement and enrolment.

Additionally, the course material for a nutrition-specific D&I science training should incorporate TPB correlation findings described here to alter intentions into behaviour change^(2,29)^. To do so, the training could use active learning strategies. Ultimately, active learning asks participants to engage by practising skills, solving problems, proposing solutions and explaining ideas. To illustrate, training participants would engage in active learning by *practising skills* (teach students through online lectures and guided readings how to apply competency-based D&I training material to nutrition interventions), *solving problems* (students conduct individual research and case studies on a poorly implementation nutrition intervention to devise solutions through D&I science frameworks), *proposing solutions* (students develop an implementation plan for an evidence-based nutrition intervention) and *explaining ideas* (students engage in reflection of course content, interact with feedback and discuss with other students). Current research demonstrates advantages of active learning strategies in D&I science instruction including increased understanding of barriers to care and improved knowledge, confidence and skills about the implementation process^(26,41,42)^. Therefore, incorporating active learning strategies provides a potential approach to addressing the TPB correlations from this study to alter D&I science intentions into behaviour change among nutrition practitioners.

### Limitations

While this study highlights potential theoretical foundations of a nutrition-specific D&I training, which is a critical need in the literature, the low response rate influences the generalisability of the findings and sampling bias. The cross-sectional design was an attempt to engage as many nutrition educators, practitioners and academics as possible. However, this design can contribute to more participation from biased individuals. Therefore, the reported results are formative and not generalisable. Likewise, the small sample size and categorisation of data made the Steel Dwass findings difficult to interpret due to the small cell sizes; however, relationships were still found to be statistically significant. Lastly, an expert is defined as someone with comprehensive knowledge in a particular area that is not retained by most colleagues, yet measuring this is subjective and vague. This led to a self-ranked measure of expertise for this study.

## Conclusions

The absence of a nutrition-specific D&I science training creates a significant knowledge capacity barrier among professionals and delays their participation, which ultimately impacts the effectiveness of interventions at changing patient-level behaviours. Fortunately, the findings from this research suggest that future nutrition-specific D&I training interventions could target perceived behavioural control (self-efficacy, knowledge and ability) through a variety of active learning strategies. While, also, taking attituded and normative beliefs into consideration by pre-evaluating and grouping individuals for training.

In addition, this research suggests that perceived behavioural control and attitudes are correlated with more experts in the field, which is key at building early adopters. To increase usage of D&I science in nutrition, more individuals need to be trained to generate more knowledgeable early adopters to ultimately build a web of programme champions, which will shift normative beliefs. To shift organisational environments, it is required to build more encouraging and self-efficient supervisors, programme champions and experts. Therefore, the implication of this formative research provides a theoretical prediction of behaviour change intention that directs educational messaging and suggests that trainings geared at perceived behavioural control will enhance self-efficacy and knowledge, increase attitudes and available trainings to ultimately create more experts in the field that will build capacity to utilise D&I science.

## Supporting information

Walker et al. supplementary material Walker et al. supplementary material

## Supplementary material

For supplementary material accompanying this paper visit https://doi.org/10.1017/S1368980023002525
