# Supplementary material for: Application of the Theory of Planned Behaviour to inform development of a Dissemination and Implementation science training for nutrition practitioners
Source: Public Health Nutr. 2023 Nov 10;26(12):3202–10. doi: 10.1017/S1368980023002525 (PMC10755436; doi:10.1017/S1368980023002525)
Supplement: Walker et al. supplementary material [file S1368980023002525sup001.docx]

Perceptions

Start of Block: BLOCK 1 - GENERAL INFORMATION

| Page Break |  |
| --- | --- |

Q1
**Contact Information**
 
**BLINDED FOR REVIEW**

Q2 Study 2.1 consent document

| 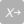 |
| --- |

Q3 **Please select a response:**

- I voluntarily agree to participate and understand that I can stop participating at anytime. (1)
- I do not agree to participate. (2)

Skip To: End of Survey If Agreement = I do not agree to participate.

End of Block: BLOCK 5 - OMR Cover Letter - No signature

Start of Block: Demographics

Q4 Please keep these definitions in mind when answering the survey Key Survey Description Definitions: 
Dissemination- Focus on “how, when, by whom, and under what circumstances research evidence spreads throughout the agencies, organizations, and front-line workers providing public health and clinical services.”    
Implementation- is the “scientific study of methods to promote the integration of research findings and evidence-based interventions into healthcare practice and policy.” It seeks to understand the behavior of those who deliver, choose, or fund interventions, as they are key variables in the adoption, implementation and sustainability of evidence-based interventions and guidelines.

Q5 What is your age?

________________________________________________________________

| 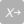 |
| --- |

Q6 What is your self-identified gender?

- Man (1)
- Woman (2)
- Non-binary / Gender nonconforming (3)
- Prefer not to disclose (4)
- prefer to self-describe (5) ________________________________________________

| 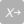 |
| --- |

Q7 Are you of Hispanic, Latino, or Spanish origin?

- Yes (1)
- No (2)

| 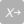 |
| --- |

Q8 Which of the following best describe your race/ethnicity? Select all that apply.

- White/Caucasian (1)
- Hispanic (2)
- Black/African American (3)
- Native American or Alaska Native (4)
- Asian/ Asian American (East Asian) (5)
- Asian/ Asian American (South Asian) (6)
- Native Hawaiian or Pacific Islander (7)
- Middle Eastern (8)
- Other/Preferred Terminology (please specify) (9) ________________________________________________

| 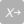 |
| --- |

Q9 What is the highest level of education you have completed?

- Less than 9th grade (1)
- 9th-11th (including 12th grade with no diploma) (2)
- High school graduate (3)
- GED or equivalent (4)
- Some college with no degree (5)
- Associates degree (6)
- Bachelor's degree (7)
- Master’s degree (8)
- Professional degree (e.g., MD, DDS, DVM, JD) (9)
- Doctorate (e.g., PhD, EdD) (10)

| 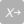 |
| --- |

Q10 Are you a registered dietitian nutritionist (RD or RDN)?

- Yes (1)
- No (2)

Q11 What is your primary profession or occupation?

________________________________________________________________

________________________________________________________________

________________________________________________________________

________________________________________________________________

________________________________________________________________

Q12 What is your area of expertise within your profession or occupation?

________________________________________________________________

________________________________________________________________

________________________________________________________________

________________________________________________________________

________________________________________________________________

Q13 Have you had any prior training in dissemination and implementation science? Please describe.

________________________________________________________________

________________________________________________________________

________________________________________________________________

________________________________________________________________

________________________________________________________________

End of Block: Demographics

Start of Block: Expert Review

| 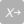 |
| --- |

Q14 How would you describe your level of expertise for dissemination and implementation science?

- **Inexperienced** (never heard of dissemination and implementation science before) (1)
- **Novice** (a person new to or inexperienced in the D&I field (i.e. a limited understanding of what D&I science is) (2)
- **Beginner** (a person just starting to learn D&I science (i.e. you have some knowledge base for example you could vaguely define what D&I science is) (3)
- **Proficient** (competent or skilled in doing or using something (i.e. you have conducted D&I research and/or attended webinars/trainings/conference sessions, and you could explain to others the many attributes of D&I science) (4)
- **Expert** (displaying special skill or knowledge derived from training or experience (i.e. you have numerous experiences conducting D&I research and/or attended many webinars/trainings/conference sessions, and you could confidently explain/teach others the many attributes of D&I science) (5)

| Page Break |  |
| --- | --- |

Q15 Questions below will ask you about your perceptions and usage of dissemination and implementation in the field of nutrition.

Q16 Please keep these definitions in mind when answering the survey. 


Key Survey Description Definitions: 


Dissemination- Focus on “how, when, by whom, and under what circumstances research evidence spreads throughout the agencies, organizations, and front-line workers providing public health and clinical services.” 


Implementation- is the “scientific study of methods to promote the integration of research findings and evidence-based interventions into healthcare practice and policy.” It seeks to understand the behavior of those who deliver, choose, or fund interventions, as they are key variables in the adoption, implementation and sustainability of evidence-based interventions and guidelines.

|  |
| --- |

Q17 I expect to use dissemination and implementation strategies (i.e. evidence-based information, evaluation tools, implementation theories and frameworks (for example: Theory of Planned Behavior, Health Belief Model, CFIR, RE-AIM, PRECEDE-PROCEED Model) to design, plan, implement, and evaluate nutrition education interventions.

- strongly disagree (1)
- disagree (2)
- somewhat disagree (3)
- neither agree nor disagree (4)
- somewhat agree (5)
- agree (6)
- strongly agree (7)

|  |
| --- |

Q18 I want to use dissemination and implementation strategies (i.e. evidence-based information, evaluation tools, implementation theories and frameworks (for example: Theory of Planned Behavior, Health Belief Model, CFIR, RE-AIM, PRECEDE-PROCEED Model) to design, plan, implement, and evaluate nutrition education interventions.

- strongly disagree (1)
- disagree (2)
- somewhat disagree (3)
- neither agree nor disagree (4)
- somewhat agree (5)
- agree (6)
- strongly agree (7)

Q19 I intend to use dissemination and implementation strategies (i.e. evidence-based information, evaluation tools, implementation theories and frameworks (for example: Theory of Planned Behavior, Health Belief Model, CFIR, RE-AIM, PRECEDE-PROCEED Model) to design, plan, implement, and evaluate nutrition education interventions.

- strongly disagree (1)
- disagree (2)
- somewhat disagree (3)
- neither agree nor disagree (4)
- somewhat agree (5)
- agree (6)
- strongly agree (7)

Q20 Using dissemination and implementation strategies (i.e. evidence-based information, evaluation tools, implementation theories and frameworks (for example: Theory of Planned Behavior, Health Belief Model, CFIR, RE-AIM, PRECEDE-PROCEED Model) to design, plan, implement, and evaluate nutrition education interventions is...

|  | 1 | 2 | 3 | 4 | 5 | 6 | 7 |  |
| --- | --- | --- | --- | --- | --- | --- | --- | --- |
|  | 1 (1) | 2 (2) | 3 (3) | 4 (4) | 5 (5) | 6 (6) | 7 (7) |  |
| Unhelpful |  |  |  |  |  |  |  | Helpful |
| Confusing |  |  |  |  |  |  |  | Clarifying |
| Ineffective |  |  |  |  |  |  |  | Effective |
| Worthless |  |  |  |  |  |  |  | Useful |
| Unimportant |  |  |  |  |  |  |  | Important |
| Burdensome |  |  |  |  |  |  |  | Easy |

Q21 What do you think your fellow nutrition colleagues expect you to do regarding using evidence-based dissemination and implementation concepts?

________________________________________________________________

________________________________________________________________

________________________________________________________________

________________________________________________________________

________________________________________________________________

Q22 How important is it that your nutrition colleagues approve of you using evidence-based dissemination and implementation concepts?

________________________________________________________________

________________________________________________________________

________________________________________________________________

________________________________________________________________

________________________________________________________________

Q23 To what extent do you think people think you should practice implementation strategies (i.e. evidence-based information, evaluation tools, implementation theories and frameworks)? 

________________________________________________________________

________________________________________________________________

________________________________________________________________

________________________________________________________________

________________________________________________________________

Q24 How motivated are you to use implementation strategies (i.e. evidence-based information, evaluation tools, implementation theories and frameworks)?

________________________________________________________________

________________________________________________________________

________________________________________________________________

________________________________________________________________

________________________________________________________________

Q25
Think about what your colleagues do when it comes to Dissemination and Implementation strategies. Then, please rate how much each apply to you by rating how much you agree or disagree.

|  | Strongly disagree (1) | Disagree (2) | Somewhat disagree (3) | Neither agree nor disagree (4) | Somewhat agree (5) | Agree (6) | Strongly agree (7) |
| --- | --- | --- | --- | --- | --- | --- | --- |
| Most colleagues who are important to me use Dissemination and Implementation strategies in their program development or interventions. (1) |  |  |  |  |  |  |  |
| Most colleagues whose opinions I value use Dissemination and Implementation strategies in their program development or interventions. (2) |  |  |  |  |  |  |  |
| Most colleagues who I care about use Dissemination and Implementation strategies in their program development or interventions. (3) |  |  |  |  |  |  |  |

Q26
Think about what your colleagues think when it comes to using Dissemination and Implementation strategies. Then, please rate how much each apply to you by rating how much you agree or disagree.

|  | Strongly disagree (1) | Disagree (2) | Somewhat disagree (3) | Neither agree nor disagree (4) | Somewhat agree (5) | Agree (6) | Strongly agree (7) |
| --- | --- | --- | --- | --- | --- | --- | --- |
| Most colleagues who are important to me **think I should** use Dissemination and Implementation strategies in my program development or interventions. (1) |  |  |  |  |  |  |  |
| Most colleagues who are important to me **expect me to** use Dissemination and Implementation strategies in my program development or interventions. (2) |  |  |  |  |  |  |  |
| **It is expected of me** that I use Dissemination and Implementation strategies in my program development or interventions. (3) |  |  |  |  |  |  |  |

Q27
Think about what your colleagues think when it comes to using Dissemination and Implementation strategies. Then, please rate how much each apply to you by rating how much you agree or disagree

|  | Strongly disagree (1) | Disagree (2) | Somewhat disagree (3) | Neither agree nor disagree (4) | Somewhat agree (5) | Agree (6) | Strongly agree (7) |
| --- | --- | --- | --- | --- | --- | --- | --- |
| Most colleagues who are important to me would **approve of me** using Dissemination and Implementation strategies in my program development or interventions. (1) |  |  |  |  |  |  |  |
| Most colleagues who are important to me would **be in favor of me** using Dissemination and Implementation strategies in my program development or interventions. (2) |  |  |  |  |  |  |  |
| Most colleagues who are important to me would **support me** in using Dissemination and Implementation strategies in my program development or interventions. (3) |  |  |  |  |  |  |  |

Q28 The last set of questions will ask you about your current level of control, knowledge, skills, and abilities to use dissemination and implementation strategies in nutrition education.

Q29 Think about your ability to use dissemination and implementation Strategies, and rate how much you agree or disagree with each statement about yourself.

|  | Strongly disagree (1) | Disagree (2) | Somewhat disagree (3) | Neither agree nor disagree (4) | Somewhat agree (5) | Agree (6) | Strongly agree (7) |
| --- | --- | --- | --- | --- | --- | --- | --- |
| I am able to use Dissemination and Implementation Strategies (1) |  |  |  |  |  |  |  |
| I am certain that I can use Dissemination and Implementation Strategies. (2) |  |  |  |  |  |  |  |
| I am confident in my ability to use Dissemination and Implementation Strategies. (3) |  |  |  |  |  |  |  |

Q30 Using Dissemination and Implementation strategies (i.e., evidence-based information, evaluation tools, implementation theories and frameworks) in developing a nutrition intervention is…

|  | Strongly disagree (1) | Disagree (2) | Somewhat disagree (3) | Neither agree nor disagree (4) | Somewhat agree (5) | Agree (6) | Strongly agree (7) |
| --- | --- | --- | --- | --- | --- | --- | --- |
| Up to me (1) |  |  |  |  |  |  |  |
| Easy for me to do. (2) |  |  |  |  |  |  |  |
| Under my control. (3) |  |  |  |  |  |  |  |

Q31 What factors or circumstances enable you to currently use D&I strategies (i.e. evidence-based information, evaluation tools, implementation theories and frameworks) in developing a nutrition intervention?

________________________________________________________________

________________________________________________________________

________________________________________________________________

________________________________________________________________

________________________________________________________________

Q32 What factors or circumstances make it difficult or impossible for you to currently use D&I strategies (i.e. evidence-based information, evaluation tools, implementation theories and frameworks) in developing a nutrition intervention?

________________________________________________________________

________________________________________________________________

________________________________________________________________

________________________________________________________________

________________________________________________________________

Q33 Are there any other issues that come to mind when you think about in using D&I strategies (i.e. evidence-based information, evaluation tools, implementation theories and frameworks) in developing a nutrition intervention?

________________________________________________________________

________________________________________________________________

________________________________________________________________

________________________________________________________________

________________________________________________________________

Start of Block: Incentive

Q34 Thank you for completing this survey. Your participation is valued! To be entered in the raffle to win one of two $75 dollar gift cards please provide your email address below.

________________________________________________________________

________________________________________________________________

________________________________________________________________

________________________________________________________________

________________________________________________________________

End of Block: Incentive
